# Light exercise heart rate on‐kinetics: a comparison of data fitted with sigmoidal and exponential functions and the impact of fitness and exercise intensity

**DOI:** 10.14814/phy2.13312

**Published:** 2017-06-22

**Authors:** Karl M. Trounson, Spencer Roberts, Aaron Balloch, Stuart A. Warmington

**Affiliations:** ^1^ Institute for Physical Activity and NutritionSchool of Exercise and Nutrition SciencesDeakin UniversityBurwoodVictoriaAustralia; ^2^ School of Medical and Health SciencesEdith Cowan UniversityPerthWestern Australia; ^3^ Fremantle Dockers Football ClubPerthWestern Australia

**Keywords:** Curve fitting, exercise training load, Heart rate, kinetics, recovery

## Abstract

This study examined the suitability of sigmoidal (SIG) and exponential (EXP) functions for modeling HR kinetics at the onset of a 5‐min low‐intensity cycling ergometer exercise test (5MT). The effects of training status, absolute and relative workloads, and high versus low workloads on the accuracy and reliability of these functions were also examined. Untrained participants (UT_abs_; *n* = 13) performed 5MTs at 100W. One group of trained participants (*n* = 10) also performed 5MTs at 100W (ET_abs_). Another group of trained participants (*n* = 9) performed 5MTs at 45% and 60% $\dot{V}O_{2}$ max (ET_45_ and ET_60_, respectively). SIG and EXP functions were fitted to HR data from 5MTs. A 30‐s lead‐in time was included when fitting SIG functions. Functions were compared using the standard error of the regression (SER), and test‐retest reliability of curve parameters. SER for EXP functions was significantly lower than for SIG functions across all groups. When residuals from the 30‐s lead‐in time were omitted, EXP functions only outperformed SIG functions in ET_60_ (EXP, 2.7 ± 1.2 beats·min^−1^; SIG, 3.1 ± 1.1 beats·min^−1^: *P* < 0.05). Goodness of fit and test–retest reliability of curve parameters were best in ET_60_ and comparatively poor in UT_abs_. Overall, goodness of fit and test–retest reliability of curve parameters favored functions fitted to 5MTs performed by trained participants at a high and relative workload, while functions fitted to data from untrained participants exercising at a low and absolute workload were less accurate and reliable.

## Introduction

In both athletic and clinical populations there is no simple and reliable measure of an individual's response to training in order to inform the immediate and long‐term training adjustments required to optimize performance (Buchheit 2014). Heart rate (HR) indices such as resting HR, HR variability, and postexercise HR recovery have received some use for this purpose (Buchheit 2014; Bellenger et al. 2016). Recently, studies have sought to model HR on‐kinetics at the onset of constant load exercise in order to estimate HR acceleration, and thus provide a level of assessment of the ability of the autonomic nervous system to rapidly meet the hemodynamic demands of exercise (Hettinga et al. 2014; Nelson et al. 2014; Thomson et al. 2016). Commonly, an exponential curve type is used to model the HR response at the onset of exercise, with monophasic functions used for trained individuals exercising at intensities up to 60% of $\dot{V}O_{2}$ max (Krzeminski et al. 1991; Feroldi et al. 1992). At higher exercise intensities biphasic models may be more suitable due to a rising sympathetic contribution following initial vagal withdrawal (Feroldi et al. 1992). Recently, the accuracy of sigmoidal curves fitted to HR data leading up to, and during, steady‐state exercise has also been investigated, with the aim of providing a more accurate estimation of autonomic responsiveness (Thomson et al. 2016).

Cross‐sectional studies have shown that trained athletes have a more rapid HR acceleration (i.e., time constant) at the onset of exercise than sedentary controls (Feroldi et al. 1992; Winlove et al. 2010; McNarry et al. 2011). Moreover, in triathletes the acute fatigue induced by a single 2‐h training session has been shown to decrease the maximal rate of HR increase (rHRI) estimated from the first derivative of both exponential and sigmoidal functions (Thomson et al. 2016). Perhaps more importantly, in cyclists and triathletes the change in the rHRI following 2 weeks of increased training load has been shown to correlate with the change in the performance of a 5‐min cycling time trial (Nelson et al. 2014; Bellenger et al. 2015). However, notwithstanding these findings limited research has been undertaken to determine the optimal test characteristics and methods of analysis of HR acceleration data obtained from the onset of exercise. While previous research has demonstrated that only the rHRI derived from a sigmoidal curve tracked changes in exercise performance when controlling for differences in baseline HR (Thomson et al. 2016), the reliability and goodness of fit of exponential and sigmoidal functions remain equivocal, while the influence of individual training status and the relative workload of the exercise stimulus have not been examined (Nelson et al. 2014; Bellenger et al. 2015; Thomson et al. 2016).

A previous investigation of rHRI test–retest reliability demonstrated some disparity between sigmoidal and exponential functions (Thomson et al. 2016). When HR data from 13 trained male cyclists were fitted to a sigmoidal function, the coefficient of variation (CV) for rHRI was 6.3% for a 5‐min 100W cycling test (Nelson et al. 2014; Thomson et al. 2016). However, a subsequent analysis of exponential functions fitted to HR data from a group of 14 male triathletes demonstrated a CV for rHRI of 13.6% (Thomson et al. 2016). Several different metrics can be used to determine goodness of fit, including the coefficient of determination (r^2^), the mean square error (MSE), and the standard error of the regression (SER) (Bitondo et al. 2011). Indeed, the data collected for the 5‐min 100 W cycle test from triathletes showed on average that a sigmoidal function produced a higher r^2^ than the exponential function, despite no difference in the MSE (Thomson et al. 2016). However, there are questions regarding the validity of the use of r^2^ for nonlinear regressions (Spiess and Neumeyer 2010). Critically, in nonlinear regressions the assumption that the total sum of squares is equal to the explained sum of squares plus the residual sum of squares is not met (Anderson‐Sprecher 1994). In addition, the previous comparison of functions included HR data during a 30‐s lead‐in period to the 5‐min cycle test when fitting with sigmoidal functions but not when fitting with exponential functions. The result was markedly different mean HR values throughout the recording period between the two function types and greater total sum of squares and favorable r^2^ values in sigmoidal functions (Thomson et al. 2016). In comparing such functions it may instead be advantageous to consider the use of SER, which represents the standard deviation of data about the regression line and is measured in the same units as the independent variable (i.e., HR in beats·min^−1^) (Manache and Melching 2008; Bitondo et al. 2011). This approach permits more informative comparisons of sigmoidal and exponential functions, as the limitations of comparing functions fitted to HR data with differing means are overcome.

With regard to the test characteristics, prior studies only examined HR on‐kinetics for a cycling test with an absolute workload (100 W) without consideration of fitness and subsequent use of a relative workload (Nelson et al. 2014; Bellenger et al. 2015; Thomson et al. 2016). While the influence of individual training status and the relative workload of the exercise stimulus has been speculated (Bellenger et al. 2015), it has not been experimentally examined. Research also suggests that there may be merit in using a greater workload for such tests given that reductions in the rHRI have been more strongly associated with performance when measured during a 5‐min treadmill running test performed at a higher relative intensity (Bellenger et al. 2015). Similarly, when comparing the HR response of marathon runners during exercise at 30% and 60% of maximum oxygen uptake ($\dot{V}O_{2}$ max), the HR overshoot effect was reduced at 60% of $\dot{V}O_{2}$ max, which appears to support the notion that higher intensities may be better suited for modeling the changes in HR at the onset of exercise (Feroldi et al. 1992). Determining the most accurate and reliable method to model HR kinetics at the onset of exercise is likely to have relevance in applied scenarios where knowledge of changes in HR kinetics may provide a basis for real‐time modifications of athlete training load.

While evidence suggests using a sigmoidal function and a test exercise intensity that is relatively high to model HR at the onset of exercise, no study has compared the suitability of sigmoidal with exponential functions on the basis of SER and test–retest reliability of curve parameters in the context of different exercise intensities and levels of aerobic fitness. Therefore, the aims of this study were to determine the suitability of sigmoidal versus exponential functions to assess HR kinetics at the onset of exercise using SER values and reliability of curve parameters. In addition, to examine the effect of training status (fitness), use of an absolute versus relative cycling workload, and use of a high versus low cycling workload on these functions for modeling HR kinetics at exercise onset.

## Methods

### Participants

A total of 32 male participants were recruited to participate in this study. Of these, 13 were untrained (UT, 23 ± 2 year, 181.7 ± 5.3 cm, 76.5 ± 6.8 kg, 51 ±5 mL·min^−1^·kg^−1^, mean ± SD) and 19 were endurance trained (ET, 28 ± 6 year, 180.6 ± 8.3 cm, 76.1 ± 8.7 kg, 60 ± 6 ml·min^−1^·kg^−1^, mean ± SD). Participants were considered endurance trained if involved in competitive cycling or triathlon training on at least 3 days per week. UT participants had not undertaken a structured exercise program for at least the previous 6 months. Prior to inclusion, participants provided written informed consent and completed a prescreening health questionnaire. Participants were excluded if presenting with musculoskeletal or neurological injury, vascular disease, or if currently taking prescribed medication for blood pressure control. The study was approved by the Deakin University Human Ethics Advisory Group.

### Study design

**Figure 1:**
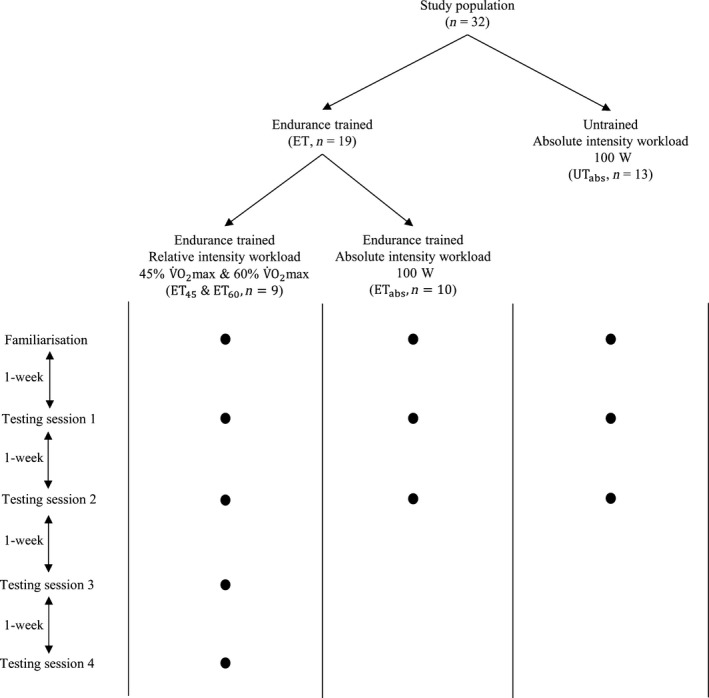
Overview of study design.

A diagrammatic representation of the study design is shown in Figure 1. All participants were required to attend the laboratory for one familiarization session and two testing sessions, each separated by 1 week. A randomly assigned subset of ET participants (*n *=* *9, 28 ± 7 year, 180.4 ± 10 cm, 77.2 ± 10.7 kg, 58 ± 6 mL·min^−1^·kg^−1^, mean ± SD) subsequently completed an additional two testing sessions (four sessions in total), each separated by 1 week. Participants were asked to refrain from consuming caffeine and alcohol on the day of each session, and from vigorous exercise in the 48‐h preceding each session. The familiarization session comprised measurements of anthropometric variables (height and body mass), after which an incremental cycling test to volitional exhaustion on an electronically braked cycle ergometer (Excalibur Sport, Lode; Groningen, the Netherlands) controlled by a computer‐running Lode Ergometry Manager software (LEM 9.3.1.0 Lode B.V., Groningen, The Netherlands) was performed to determine maximal oxygen uptake ($\dot{V}O_{2}$ max) and ventilatory threshold (VT).

Each testing session required participants to complete a 5‐min low‐intensity exercise test (5MT) on the cycle ergometer. UT (*n* = 13) performed the 5MT at an absolute intensity of 100 W (UT_abs_). ET participants were randomly assigned to perform the 5MT at either an absolute intensity of 100 W (ET_abs_, *n *=* *10, 29 ± 5 year, 180 ± 6.7 cm, 74.7 ± 5.8 kg, 62 ± 6 mL·min^−1^·kg^−1^, mean ± SD) or at relative intensities of both 45% $\dot{V}O_{2}$ max and 60% $\dot{V}O_{2}$ max (ET_45_ and ET_60_, respectively, *n *=* *9, 28 ± 7 year, 180.4 ± 10 cm, 77.2 ± 10.7 kg, 58 ± 6 mL·min^−1^·kg^−1^, mean ± SD), thus completing two tests at each intensity. These relative intensities were chosen given that 45% $\dot{V}O_{2}$ max for trained individuals approximates a workload of 100 W, while 60% $\dot{V}O_{2}$ max elicits a sufficient sympathetic response to limit the HR overshoot phenomenon that occurs with submaximal intensity exercise when there is a feeble sympathetic response (Feroldi et al. 1992). The HR overshoot effect results in a notch in the HR on‐response at the beginning of exercise, making it less conducive to a monoexpontential curve fitting process (Feroldi et al. 1992). For ET_45_ and ET_60_, 5MT intensity was randomized to minimize the influence of learning effects over the four testing sessions on outcome measures. HR was continuously recorded throughout the testing session on a beat‐to‐beat basis (RS800cx, Polar Electro; Kemple, Finland) for subsequent analysis of the HR kinetic response to the 5MT.

### Procedure

#### Incremental cycling test to exhaustion

The incremental cycling test to exhaustion commenced at a workload of 75 W and increased by 50 W every 3 min. After 9 min, workload increased by 25 W every 1 min until volitional fatigue. Breath‐by‐breath gas exchange was measured throughout the test using an Innocor metabolic system (DK‐5260, Innovision, Odense, Denmark) to determine $\dot{V}O_{2}\;\text{max}.$ VT was determined using the V‐slope method (Beaver et al. 1986).

#### 5MT

The 5MT required participants to sit resting on the cycle ergometer for a 30‐s period and then cycle at a predetermined power output for 5 min. The cycle ergometer was set to pedal rate independent mode and participants were allowed to select their own cadence within the range 80–100 rpm. Participants were unaware of the starting time of each 5MT so as to avoid an anticipatory increase in HR prior to the test (Krogh and Lindhard 1913).

### Data management and statistical analyses

#### HR kinetics

Beat‐to‐beat HR data recorded during each 5MT were transferred to Table Curve 2D software (SYSTAT Software Inc., San Jose, California, USA) and fitted to a sigmoidal function according to equation (1) (SIG), and a monoexponential function according to equation (2) (EXP) using a nonlinear least squares approach.

(1) $$\text{HR}\left(\text{beats}\cdot {\text{min}}^{-1}\right)=a+\left(\frac{A}{1+e^{-\tau_{\mathrm{SIG}}\left(t-HR_{50}\right)}}\right)$$

(2) $$\text{HR}\left(\text{beats}\cdot {\text{min}}^{-1}\right)=a+A\left(1-e^{\frac{-(t-\mathrm{TD})}{\tau_{\mathrm{EXP}}}}\right)$$

*a,* baseline HR value (beats·min^−1^); *A*, amplitude of HR response (beats·min^−1^); t, time (s); HR_50_, time at which half of HR response amplitude was reached (s); TD, time delay before HR increases sharply (s); *τ*_SIG_, SIG function curvature parameter (s); *τ*_EXP,_ EXP function curvature parameter (s).

Equations (1) and (2) were inputted as user‐defined functions in the Table Curve 2D software equation set. For SIG, beat‐to‐beat HR data from the 30‐s prior to the commencement of the 5MT were included in the 5MT HR data and were included in the curve fitting process. For both the SIG and EXP functions graphical adjustment was performed prior to fitting to determine appropriate starting estimates and constraints for each parameter (Findlay and Dillard 2007). Where the initial fitting process yielded a baseline HR value outside a range encompassed by ±1.96 SD from the average HR during the 30‐s resting period prior to the commencement of cycling, baseline HR was constrained to fit within this range. For EXP functions, where the initial fitting process yielded a TD value outside the range 0–5 s, the TD was then constrained to fit within this range as this is the typical TD range for the HR response to increases in workload (Broman and Wigertz 1971; Miyamoto et al. 1982).

The rHRI (beats·min^−1^·s^−1^) was determined from the first derivative maxima from the SIG and EXP functions according to equation (3) and equation (4), respectively.

(3) $$\text{rHRI}\left(\text{beats}\cdot {\text{min}}^{-1}\cdot s^{-1}\right)=\frac{A\left(\tau_{\mathrm{SIG}}\left(e^{-\tau_{\mathrm{SIG}}\left(t-HR_{50}\right)}\right)\right)}{{\left(e^{-\tau_{\mathrm{SIG}}\left(t-HR_{50}\right)}+1\right)}^{2}}$$

(4) $$\text{rHRI}\left(\text{beats}\cdot {\text{min}}^{-1}\cdot s^{-1}\right)=A\frac{\left(e^{-1\left(\frac{t-\mathrm{TD}}{\tau_{\mathrm{EXP}}}\right)}\right)}{\tau_{\mathrm{EXP}}}$$

#### Statistical analyses

All data are expressed as mean ± SD unless otherwise stated. The goodness of fit for the SIG and EXP functions fitted to 5MT HR data was assessed using the standard error of the regression (SER). SER was calculated by taking the square root of the mean square error (MSE) of each function fitted to HR data. A time‐adjusted SER was also determined for SIG functions by excluding residuals during the 30 s of the 5MT prior to commencement of exercise to allow for more meaningful comparison of the goodness of fit between SIG and EXP functions. Within‐group comparisons of SER, time‐adjusted SER, baseline HR, HR amplitude, and rHRI values between SIG and EXP functions were performed using a paired sample *t*‐test. The curve parameters HR_50_, TD, *τ*_SIG_, and *τ*_EXP_ were excluded from within‐group comparisons, as these parameters were not common across SIG and EXP functions. Prior to paired sample *t*‐test analysis, the distribution of each variable was examined with a Shapiro–Wilk normality test. In instances where data were skewed a log transformation was performed to allow parametric statistical comparison. Between‐group comparisons of SER, time‐adjusted SER, curve parameters, and rHRI values for SIG and EXP functions were performed using a one‐way ANOVA, except in the case of comparisons between ET_45_ and ET_60_ data, where a repeated‐measures one‐way ANOVA was used. Significant effects were examined using the Tukey–Kramer post hoc test. Data that violated the Levene test of homogeneity were log transformed prior to analysis.

Coefficients of variation (CV) and Bland–Altman's limits of agreement (LoA) (±1.96 SD) were calculated for SER, time‐adjusted SER, curve parameters, and rHRI for SIG and EXP functions fitted to 5MT HR data to assess test–retest reliability.

## Results

### Incremental cycling test to exhaustion

5MT cycling workloads, $\dot{V}O_{2}$ max, and VT data for all groups are shown in Table 1. Fitness ($\dot{V}O_{2}$ max) and VT were greater for all ET groups compared with UT_abs_, and while 5MT cycling workloads were similar between UT_abs_, ET_abs_, and ET_45_, these were all lower than for ET_60_.

**Table 1:** $\dot{V}O_{2}$ max, VT, and workload data from incremental cycling test to exhaustion

Group	$\dot{V}O_{2}$ max (mL·min^−1^·kg^−1^)	VT (mL·min^−1^·kg^−1^)	Workload (W)
UT_abs_	51 ± 5	39 ± 4	100 ± 0
ET_abs_	62 ± 6^a^	50 ± 5^a^	100 ± 0
ET_45_	58 ± 6^a^	48 ± 8^a^	104 ± 13
ET_60_	58 ± 6^a^	48 ± 8^a^	162 ± 19^b^

All values expressed as mean ± SD. UT_abs_: Untrained, absolute intensity; ET_abs_: Endurance trained, absolute intensity; ET_45_: Endurance trained, 45% $\dot{V}O_{2}$ max; ET_60_: Endurance trained, 60% $\dot{V}O_{2}$ max.

a Significant difference versus UT_abs_ (*P* < 0.05).

b Significant difference versus UT_abs_, ET_abs_, and ET_45_ (*P* < 0.05).

### Goodness of fit

#### SER

SER was significantly lower for EXP compared with SIG in all groups (*P* < 0.05) (Table 2). SER for UT_abs_ was significantly greater than all other groups for both SIG and EXP (SIG, 6.5 ± 1.7 beats·min^−1^; EXP, 6.1 ± 1.6 beats·min^−1^: *P* < 0.05). Only for EXP was SER significantly lower for ET_60_ (2.7 ± 1.2 beats·min^−1^) compared with ET_abs_ (3.9 ± 1.1 beats·min^−1^: *P* < 0.05). Test–retest reliability for SIG appeared better in ET_abs_ (17% CV, 95% LoA ‐1.8‐0.7) and ET_60_ (17% CV, 95% LoA ‐1‐1.6) compared with UT_abs_ (21% CV, 95% LoA ‐4.1‐3.5) and ET_45_ (29% CV, 95% LoA ‐2‐2.5).

**Table 2:** Goodness of fit, curve parameters, and rate of heart rate increase mean ± SD and test–retest reliability for SIG and EXP curves across all groups

	SIG			EXP		
			95% Limits of agreement			95% Limits of agreement
	Mean ± SD	CV (%)	Bias (± 1.96 SD)	Mean ± SD	CV (%)	Bias (± 1.96 SD)
SER (beats·min^−1^)						
UT_abs_	6.5 ± 1.7	21	−0.3 (3.8)	6.1 ± 1.6^a^	20	−0.1 (3.2)
ET_abs_	4.1 ± 1.1^b^	17	−0.5 (1.2)	3.9 ± 1.1^a^, ^b^	20	−0.4 (2.7)
ET_45_	3.4 ± 0.7^b^	29	0.2 (2.2)	3.2 ± 0.7^a^, ^b^	25	0.0 (2.0)
ET_60_	3.4 ± 1.1^b^	17	0.3 (1.3)	2.7 ± 1.2^a^, ^b^, ^c^	23	0.2 (1.4)
Time‐adjusted SER (beats·min^−1^)						
UT_abs_	6.4 ± 1.7	21	−0.4 (3.8)	6.1 ± 1.6	20	−0.1 (3.2)
ET_abs_	3.9 ± 1.0^b^	19	−0.4 (2.5)	3.9 ± 1.1^b^	20	−0.4 (2.7)
ET_45_	3.2 ± 0.7^b^	30	0.2 (2.4)	3.2 ± 0.7^b^	25	−0.0 (2.0)
ET_60_	3.1 ± 1.1^b^	16	0.2 (1.2)	2.7 ± 1.2^a^, ^b^, ^c^	23	0.2 (1.4)
Baseline HR (beats·min^−1^)						
UT_abs_	69 ± 14	7	−2 (24)	77 ± 16^a^	11	5 (32)
ET_abs_	66 ± 9	6	−4 (15)	65 ± 8	8	−3 (20)
ET_45_	72 ± 9	7	−6 (14)	74 ± 9	6	−3 (17)
ET_60_	72 ± 11	8	2 (19)	79 ± 9^a^, ^c^	3	1 (8)
HR amplitude (beats·min^−1^)						
UT_abs_	44 ± 11	14	6 (20)	38 ± 13^a^	24	−1 (28)
ET_abs_	30 ± 5^b^	12	3 (13)	31 ± 6	13	1 (14)
ET_45_	44 ± 9^c^	11	5 (14)	41 ± 11	13	3 (22)
ET_60_	65 ± 11^b^, ^c^, ^d^	4	−1 (9)	59 ± 8^a^, ^b^, ^c^, ^d^	9	0 (17)
HR_50_ (s)						
UT_abs_	12 ± 8	39	−4 (12)			
ET_abs_	8 ± 3	30	−1 (7)			
ET_45_	10 ± 4	23	−2 (9)			
ET_60_	16 ± 5^c^	20	2 (12)			
TD (s)						
UT_abs_				0.5 ± 1.4	121	−0.6 (4.1)
ET_abs_				0.9 ± 1.2	91	−0.1 (2.6)
ET_45_				1.1 ± 1.8	105	−0.7 (2.3)
ET_60_				0.7 ± 1.2	93	0.1 (1.8)
*τ*_SIG_ (s)						
UT_abs_	0.1 ± 0.1	30	0.0 (0.3)			
ET_abs_	0.4 ± 0.3^b^	32	−0.1 (0.9)			
ET_45_	0.2 ± 0.1	24	0.0 (0.2)			
ET_60_	0.1 ± 0.0^c^	9	0.0 (0.1)			
*τ*_EXP_ (s)						
UT_abs_				27 ± 21	51	−10.7 (49.5)
ET_abs_				8 ± 3^b^	33	−0.4 (7.1)
ET_45_				12 ± 4	15	−0.3 (7.5)
ET_60_				22 ± 6^c^	13	3.3 (8.9)
rHRI (beats·min^−1^·s^−1^)						
UT_abs_	1.5 ± 1.2	34	0.1 (2.6)	2.4 ± 2.1^a^	38	0.4 (3.7)
ET_abs_	2.5 ± 1.6	31	−0.6 (4.3)	5.4 ± 2.9^a^, ^b^	32	−0.5 (8.2)
ET_45_	2.1 ± 1.1	21	0.4 (1.8)	4.4 ± 2.5^a^	14	0.4 (2.5)
ET_60_	1.5 ± 0.4	12	−0.1 (0.7)	3 ± 1.0^a^	13	−0.5 (1.2)

All values expressed as mean ± SD unless otherwise stated. UT_abs_, Untrained, absolute intensity; ET_abs_, Endurance trained, absolute intensity; ET_45_: Endurance trained, 45% $\dot{V}O_{2}$ max; ET_60_, Endurance trained, 60% $\dot{V}O_{2}$ max; SER, Standard error of regression; HR_50_, Time taken for half of HR response amplitude to be reached; TD, Time delay before HR increases sharply; *τ*_SIG_, SIG function curvature parameter; *τ*_EXP_, EXP function curvature parameter; rHRI, Rate of heart rate increase.

a Significant difference versus SIG (*P* < 0.05).

b Significant difference versus UT_abs_ (*P* < 0.05).

c Significant difference versus ET_abs_ (*P* < 0.05).

d Significant difference versus ET_45_ (*P* < 0.05).

#### Time‐adjusted SER

For UT_abs_, ET_abs_, and ET_45_ groups, time‐adjusted SER was not different between SIG and EXP. However, for ET_60_ time‐adjusted SER was greater for SIG (3.1 ± 1.1 beats·min^−1^) compared with EXP (2.7 ± 1.2 beats·min^−1^: *P* < 0.05). Time‐adjusted SER for SIG was significantly greater in UT_abs_ (6.4 ± 1.7 beats·min^−1^) than all other groups (*P* < 0.05). Test–retest reliability for SIG appeared best in ET_60_ (16% CV, 95% LoA ‐0.99‐1.4).

### Curve parameters

#### Baseline HR

**Figure 2:**
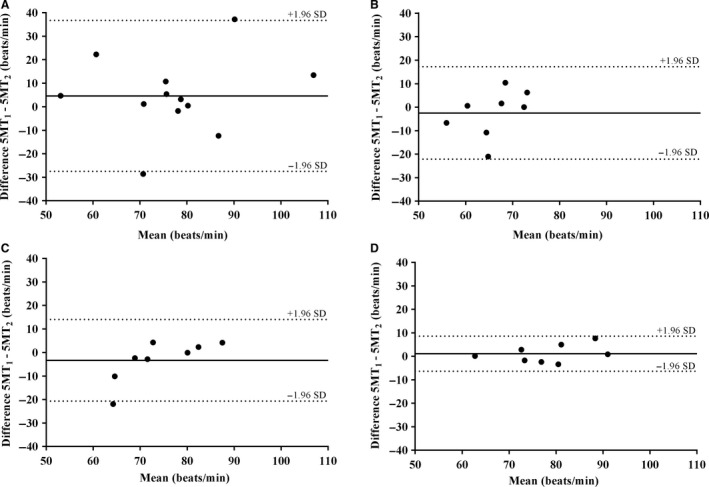
Bland–Altman analyses of baseline HR measurements from 5MT_1_ and 5MT_2_ when fitted with an exponential function (EXP) in (A) UT_abs_, (B) ET_abs_, (C) ET_45_, and (D) ET_60_. Solid lines represent the average difference between 5MT_1_ and 5MT_2_ (i.e., bias) and the dotted lines represent the upper and lower 95% confidence limits of agreement.

Baseline HR was not different between SIG and EXP for ET_abs_ and ET_45_. However, baseline HR for UT_abs_ and ET_60_ was lower for SIG than EXP (69 ± 14 beats·min^−1^ vs, 76 ± 16 beats·min^−1^ and 72 ± 11 beats·min^−1^ vs. 79 ± 9 beats·min^−1^, respectively: *P* < 0.05). Test–retest reliability for baseline HR was similar between groups for SIG. However, for EXP test–retest reliability was best in ET_60_ (3% CV, 95% LoA ‐6‐9) and poorest in UT_abs_ (11% CV, 95% LoA ‐27‐37). Individual differences in baseline HR for EXP across the two 5MTs performed in all groups are shown as Bland–Altman plots in Figure 2A–D. Test–retest reliability for UT_abs_ was better for SIG (7% CV, 95% LoA ‐26‐22) compared with EXP (11% CV, 95% LoA ‐27‐37), while for ET_60_, baseline HR test–retest reliability was better for EXP (3% CV, 95% LoA ‐6‐9) compared with SIG (8% CV, 95% LoA ‐17‐22).

#### HR amplitude

HR amplitude was not different between SIG and EXP for ET_abs_ and ET_45_. However, HR amplitude for UT_abs_ and ET_60_ was higher for SIG than EXP (44 ± 11 vs. 38 ± 13 beats·min^−1^ and 65 ± 11 vs. 59 ± 8 beats·min^−1^, respectively: *P* < 0.05). While HR amplitude for EXP was not different among UT_abs_, ET_abs_, and ET_45_, it was greater than all groups in ET_60_. However, HR amplitude for SIG was progressively greater from ET_abs_ to UT_abs_ and ET_45_, and to ET_60_.

Test–retest reliability for HR amplitude was better for SIG in UT_abs_ (14% CV, 95% LoA ‐14‐26), ET_45_ (11% CV, 95% LoA ‐9‐19), and ET_60_ (4% CV, 95% LoA ‐11‐8) than for EXP (24% CV, 95% LoA ‐29‐26; 13% CV, 95% LoA ‐18‐25; and 9%, 95% LoA ‐17‐17, respectively). Test–retest reliability was best in ET_60_ for SIG (4% CV, 95% LoA ‐11‐8) and poorest in UT_abs_ for both SIG (14% CV, 95% LoA ‐14‐26) and EXP (24% CV, 95% LoA ‐29‐26).

#### Hr_50_

HR_50_ for SIG was not different among UT_abs_ (12 ± 8 s), ET_abs_ (8 ± 3 s), and ET_45_ (10 ± 4 s), with ET_abs_ being significantly lower than ET_60_ (16 ± 5 s: *P* < 0.05). Test–retest reliability was poorest in UT_abs_ (39% CV, 95% LoA ‐16‐9).

#### TD

TD for EXP functions was not different between groups.

#### *τ*_Sig_

For SIG functions, *τ*_SIG_ was greater in ET_abs_ (0.4 ± 0.3 s) compared with UT_abs_ (0.1 ± 0.1 s) and ET_60_ (0.1 ± 0.0 s: *P* < 0.05). Test–retest reliability for *τ*_SIG_ was best in ET_60_ (9% CV, 95% LoA ‐0.1‐0.1), followed by ET_45_ (24% CV, 95% LoA ‐0.2‐0.2), UT_abs_ (30% CV, 95% LoA ‐0.3‐0.3), and then ET_abs_ (32% CV, 95% LoA ‐1.0‐0.7).

#### ***τ***_**EXP**_

For EXP functions, *τ*_EXP_ was lower in ET_abs_ (8 ± 3 s) compared with UT_abs_ (27 ± 21 s) and ET_60_ (22 ± 6 s: *P* < 0.05). Test–retest reliability for *τ*_EXP_ was best in ET_60_ and ET_45_ (13% CV, 95% LoA ‐5.6‐12.2 and 15% CV, 95% LoA ‐7.8‐7.2, respectively), followed by ET_abs_ (33% CV, 95% LoA ‐7.5‐6.7), and then UT_abs_ (51% CV, 95% LoA ‐60.2‐38.8).

### Maximal rate of HR increase

#### rHRI

The rHRI for EXP was significantly greater compared with SIG in all groups (*P* < 0.05). Test–retest reliability for rHRI was best in ET_60_ across both SIG and EXP functions (SIG, 12% CV, 95% LoA ‐0.9‐0.6; EXP, 13% CV, 95% LoA ‐1.7‐0.7). Test–retest reliability tended to be poorest in UT_abs_ (SIG, 34% CV, 95% LoA –2.5‐2.7; EXP, 38% CV, 95% LoA ‐3.3‐4.1) and ET_abs_ (SIG, 31% CV, 95% LoA ‐4.9‐3.7; EXP, 32% CV, 95% LoA ‐8.7‐7.7) across both SIG and EXP.

## Discussion

The main findings of this study when examining HR kinetic data collected at the onset of a 5‐min low‐intensity constant load cycling exercise bout (5MT) were that (1) EXP functions demonstrated superior goodness of fit to SIG functions; (2) while this difference was largely eliminated when comparing EXP and SIG functions using a time‐adjusted SER, it was still evident in trained participants undertaking the 5MT at a slightly higher exercise intensity (ET_60_); (3) goodness of fit and test–retest reliability of curve parameters tended to be favorable in functions fitted to HR data from 5MTs undertaken by trained participants at a high workload and a relative workload, while for untrained participants functions fitted to HR data demonstrated relatively poor goodness of fit and curve parameter test–retest reliability, particularly at a low workload and an absolute workload. The use of HR kinetics at the onset of exercise provides a novel approach to potentially inform about autonomic function immediately prior to exercise training or performance and these data suggest that examination of HR kinetic data from 5MTs may be more suited to trained participants when used at a high and relative workload, and when modeled using an exponential function.

### Comparison of functions

**Figure 3:**
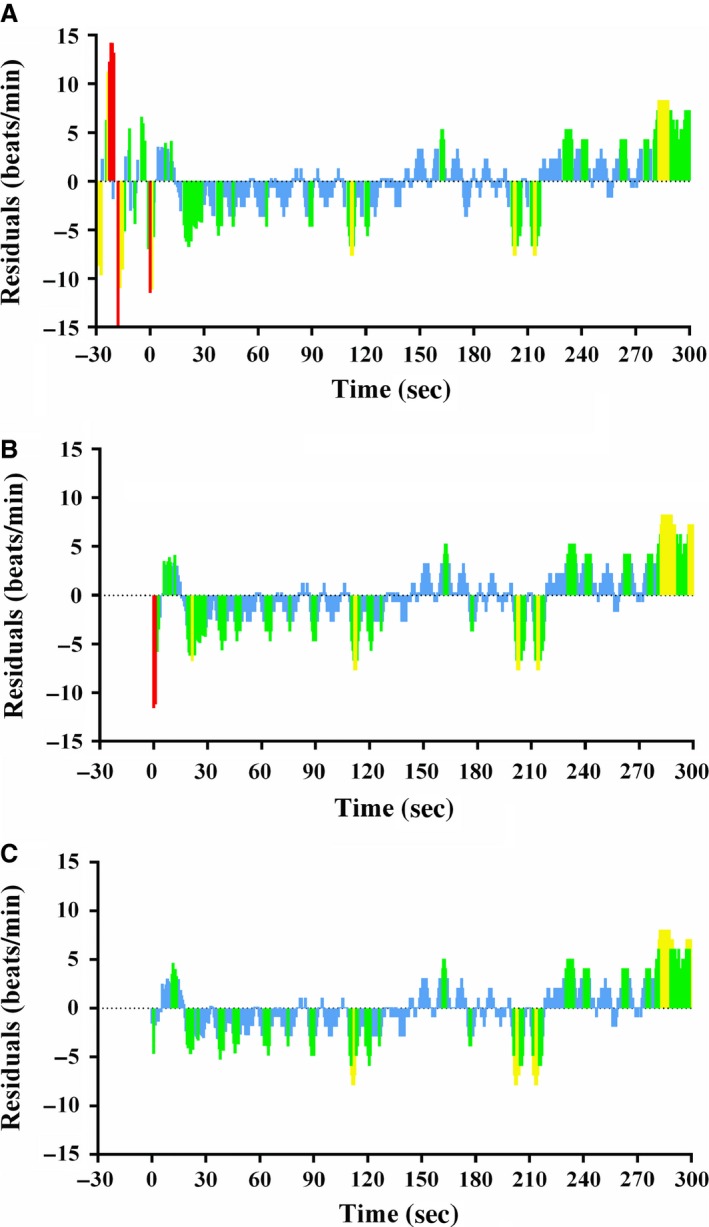
Residual plots for (A) SIG function, (B) time‐adjusted SIG function, and (C) EXP function fitted to HR data from a 5MT performed by a representative ET_45_ participant. Residuals <1 SD from the regression line are shown as blue colored bars; residuals ≥ 1 SD and < 2 SD from the regression line are shown as green colored bars; residuals ≥ 2 SD and < 3 SD from the regression line are shown as yellow colored bars; residuals ≥ 3 SD from the regression line are shown as red colored bars.

Previous studies have compared the goodness of fit of EXP and SIG functions to HR data collected at the onset of exercise using the coefficient of determination (*r* ^2^) and mean square error (MSE) (Thomson et al. 2016). This study used the square root of MSE (SER) for goodness‐of‐fit evaluation as *r* ^2^ has limitations when applied to nonlinear models and when comparing goodness of fit of functions fitted to datasets with different sample means (Spiess and Neumeyer 2010). The present study also included a comparison between the SER of EXP functions and SIG functions in which residuals during the 30 s prior to the commencement of exercise were excluded given that residual plots revealed a tendency for high fluctuations in HR during the 30‐s prior to exercise, thus causing the SER to be inflated for SIG functions. Examples of residual plots for the SIG, time‐adjusted SIG, and EXP functions fitted to HR data from one 5MT of an ET_45_ participant are shown in Figure 3A–C. However, even when comparing against the time‐adjusted SER for SIG functions, EXP functions still demonstrated superior goodness of fit at a high relative intensity in endurance‐trained participants (ET_60_). This may be explained by the higher exercise intensity resulting in a longer time until the HR plateau, as evidenced by greater HR_50_ values under this condition, as well as a greater number of data points during the plateau phase due to the shorter R‐R intervals compared with exercise at a lower intensity (Tulppo et al. 1998; Bellenger et al. 2015). These characteristics result in ET_60_ data being less conducive to a symmetrical sigmoidal function fit. Sigmoidal functions applied to data from ET_60_ favored fitting to the third inflection point (concave down) before the upper HR plateau and were compromised around the first (concave up) inflection point where there were fewer data points, especially for non‐time‐adjusted SER from SIG functions that include the 30‐s resting data prior to exercise. As such, EXP functions are able to minimize SER due to only having a single inflection point and thus make EXP functions more likely to be appropriate for fitting to HR kinetic data at the onset of exercise under most circumstances.

As well as goodness of fit, test–retest reliability of curve parameters informs the process of determining the most suitable function, training status, and exercise intensity for modeling HR kinetics at the onset of exercise. Better test–retest reliability of baseline HR and HR amplitude points to a more consistent curve fitting process. Thus, when applied practically in the field, any changes associated with fatigue or training adaptations may be more reliably identified as real, rather than being false positives. In addition, rHRI and the curvature parameters *τ*_EXP_ and *τ*_SIG_, which can also be used to characterize cardiac acceleration given that they dictate the shape of the curve, have been correlated with exercise performance and should also exhibit minimal variability between trials in order to have potential value in the assessment and management of fatigue and recovery immediately prior to exercise that may be used to inform about modifications to training load (Bunc et al. 1988; Thomson et al. 2016).

Across all exercise intensities, test–retest reliability in HR amplitude was lower when SIG functions were fitted. This is an important finding that practitioners should consider when fitting functions to HR data at lower exercise intensities where there is no difference in goodness of fit between EXP and time‐adjusted SIG functions.

rHRI test–retest reliability overall was poorer than has previously been reported (Thomson et al. 2016). However, there was no marked difference between SIG and EXP functions across all groups, which conflicts with findings from Thomson et al. (2016) who reported better test–retest reliability in rHRI derived from SIG functions. These discrepancies may have been due to slight methodological differences compared with the present study, including the software used to model functions, constraints and starting estimates applied to functions, and the use of HR data averaged over 1‐s intervals as opposed to beat‐to‐beat data used in the present study (Thomson et al. 2016). Using beat‐to‐beat data allows practitioners to compare HR on‐kinetics with heart rate variability data during rest and exercise periods, which also shows promise as an indicator of training‐induced autonomic fatigue (Buchheit 2014). Thomson et al. (2016) also reported an inverse relationship between baseline HR and rHRI, pointing to a link between the two parameters. As such, the higher CVs for rHRI in the present study may be a result of more variable baseline HR data.

### Effect of training status

Trained participants exhibited lower HR amplitude and greater rHRI compared with untrained participants exercising at commensurate intensity, which has been demonstrated in previous research (Mcardle et al. 1977; Wilmore et al. 1996; Hettinga et al. 2014). These observations have been attributed to cardiovascular adaptations, including improved oxygen transport and hemodynamics, and increased resting vagal tone among trained individuals, allowing for greater parasympathetic withdrawal and faster tachycardia at the onset of exercise (Bunc et al. 1988; Chacon‐Mikahil et al. 1998; Hettinga et al. 2014). Importantly, this study also showed that the goodness of fit of curves fitted to HR data from untrained participants was poorer, and several curve parameters including baseline HR for EXP functions, HR amplitude, HR_50_, *τ*_EXP_, and rHRI tended to show greater variability between exercise trials when compared with trained participants. While the reasons for these differences in goodness of fit and curve parameter variability remain unclear, noticeable fluctuations in HR at the onset of exercise can occur as a result of transient increases in vagal tone associated with changes in arterial pressure and the baroreceptor reflex (Fagraeus and Linnarsson 1976). Endurance‐trained individuals demonstrate inhibited arterial baroreceptor reflex activity in response to phenylephrine‐induced increases in mean arterial pressure compared with individuals of average fitness (Shi et al. 1993), which may point to greater fluctuations in HR at the onset of exercise and hence less favorable goodness of fit and curve parameter test–retest reliability among untrained individuals.

### Effect of absolute versus relative exercise intensity

There was no difference in goodness of fit of functions when data from participants exercising at an absolute intensity of 100 W were compared with that of participants exercising at a relative intensity of 45% $\dot{V}O_{2}$ max. However, test–retest reliability of key curve parameters including *τ*_EXP_, *τ*_SIG_, and rHRI was minimized under the 45% $\dot{V}O_{2}$ max condition. Therefore, exercise bouts at relative workloads may therefore be more appropriate in applied scenarios and provide greater information about autonomic responsiveness on an individual athlete basis.

Comparisons between goodness of fit of curves on the basis of intensity also revealed that EXP functions fitted to 5MTs performed at 60% $\dot{V}O_{2}$ max had superior goodness of fit compared with 5MTs performed at 100 W. This is likely due to trained individuals exhibiting lower HR variability and thus more uniform R‐R intervals conducive to a better EXP fit when exercising at higher intensities (Al Haddad et al. 2011).

### Effect of high versus low exercise intensity

While there was no difference in goodness of fit between functions fitted to 5MTs undertaken at 60% $\dot{V}O_{2}$ max compared with 45% $\dot{V}O_{2}$ max, time‐adjusted SER, HR amplitude, and *τ*_SIG_ for SIG functions, baseline HR for EXP functions, and rHRI across both functions were all more reliable at the higher intensity. It has been suggested that rHRI may better track exercise performance when 5MTs are performed at intensities greater than 100 W (Bellenger et al. 2015), and the findings of this study provide further support for the use of higher‐intensity 5MTs (e.g., 60% $\dot{V}O_{2}$ max).

### Limitations and future research

A limitation of this study was that given UT_abs_ performed the 5MT at a low, absolute intensity only, the assertion that using HR kinetics for an assessment of autonomic responsiveness to a 5MT is more accurate and reliable in a fitter population should be made with caution. It remains unclear the extent to which exercising at a higher, relative intensity improves the accuracy of HR kinetic modeling in untrained individuals.

In addition, while the highest‐intensity 5MTs gave rise to HR data that were able to be modeled most accurately, questions remain about whether functions fitted to such 5MTs will detect changes in parasympathetic modulation following a period of heavy training given that the higher‐intensity workload elicits a greater sympathetic response (Le Meur et al. 2013; Bellenger et al. 2016). Future research should seek to address this and should focus on attempting to track fatigue‐ and training‐induced changes in exercise performance using the most appropriate conditions, function, and curve parameters identified in this study.

Future research may also investigate the effects of setting relative workloads according to percentages of athletes' maximum HR, as well as $\dot{V}O_{2}$ max. Setting the intensity of 5MTs as a percentage of maximum HR may allow for easier application in the field.

In the context of previous research in which the rHRI derived from sigmoidal functions was best able to track changes in exercise performance (Thomson et al. 2016), the current findings suggest that an exponential function and its associated parameters should not be discounted as a potential method of tracking autonomic responsiveness and subsequent exercise performance, and may even be favorable based on the present curve fitting approach. In addition, the good test–retest reliability of curvature parameters and rHRI among trained individuals supports the use of these parameters, which have previously been shown to be positively related to exercise performance (Bunc et al. 1988; Nelson et al. 2014; Bellenger et al. 2015; Thomson et al. 2016).

## Conclusion

Analysis of HR kinetics at the onset of submaximal exercise appears to be a promising means by which to monitor athlete fatigue, recovery, readiness for further training and competition, and possibly fitness adaptations. This study sought to determine the exercise conditions that are most conducive to an accurate and reliable analysis of HR kinetic data with the view to providing practitioners with a methodological framework for undertaking such analysis. The level of autonomic recovery or adaptation as assessed by a HR kinetic analysis may ultimately inform modifications to training workloads prior to a training session, as well as expectations of performance and strategies with regard to player management during competition (e.g., substitutions and interchanges). This study compared functions on the basis of SER, which is a more appropriate measure of goodness of fit than r^2^ in the context of nonlinear data with varying sample means. Results showed that when 5MTs are performed at a higher intensity, that is, 60% $\dot{V}O_{2}$ max, the use of EXP functions to model HR data is particularly favorable, but likely more reliable under most 5MT exercise conditions. Using curve parameters to track adaptations or changes in performance of untrained individuals may be limited given that functions tended to fit poorly to HR data from these individuals, while curve parameters were also more variable. Curve parameters from functions fitted to HR data from 5MTs performed at a relative intensity also appear more reliable than at an absolute intensity, with functions fitted to HR from 5MTs undertaken at a high, relative intensity of 60% $\dot{V}O_{2}\;\text{max}$ providing the most reliable curve parameters. Therefore, based on these findings, practitioners using rHRI and other curve parameters as performance trackers in trained individuals should consider using curve parameters from EXP functions fitted to HR data from 5MTs performed at 60% $\dot{V}O_{2}$ max.

## Conflict of Interest

No conflicts of interest, financial or otherwise, are declared by the authors. The results are presented clearly, honestly, and without fabrication, falsification, or inappropriate data manipulation.
